# How to foster successful implementation of a patient reported experience measurement in the disability sector: an example of developing strategies in co-creation

**DOI:** 10.1186/s40900-021-00287-w

**Published:** 2021-06-24

**Authors:** Marjolein van Rooijen, Anneke van Dijk-de Vries, Stephanie Lenzen, Ruth Dalemans, Albine Moser, Anna Beurskens

**Affiliations:** 1 Department of Family Medicine, CAPHRI School for Public Health and Primary Care, Maastricht University, P. Debyeplein 1, 6229 HA Maastricht, The Netherlands; 2 Research Centre for Autonomy and Participation of Persons with a Chronic Illness, Zuyd University of Applied Sciences, Heerlen, The Netherlands

**Keywords:** Co-creation, Disability care, Participatory researcher, Implementation science, Communication-vulnerable care-user, Stakeholder engagement

## Abstract

**Background:**

The integrated uptake of patient-reported experience measures, using outcomes for the micro, meso and macro level, calls for a successful implementation process which depends on how stakeholders are involved in this process. Currently, the impact of stakeholders on strategies to improve the integrated use is rarely reported, and information about how stakeholders can be engaged, including care-users who are communication vulnerable, is limited. This study illustrates the impact of all stakeholders on developing tailored implementation strategies and provides insights into supportive conditions to involve care-users who are communication vulnerable.

**Methods:**

With the use of participatory action research, implementation strategies were co-created by care-users who are communication vulnerable (*n* = 8), professionals (*n* = 12), management (*n* = 6) and researchers (*n* = 5) over 9 months. Data collection consisted of audiotapes, reports, and researchers’ notes. Conventional content analysis was performed.

**Results:**

The impact of care-users concerned the strategies’ look and feel, understandability and relevance. Professionals influenced impact on how to use strategies and terminology. The impact of management was on showing the gap between policy and practice, and learning from previous improvement failures. Researchers showed impact on analysis, direction of strategy changes and translating academic and development experience into practice. The engagement of care-users who are communication vulnerable was supported, taking into account organisational issues and the presentation of information.

**Conclusions:**

The impact of all engaged stakeholders was identified over the different levels strategies focused on. Care-users who are communication vulnerable were valuable engaged in co-creation implementation strategies by equipping them to their needs and routines, which requires adaptation in communication, delimited meetings and a safe group environment.

**Trial registration:**

Reviewed by the Medical Ethics Committee of Zuyderland-Zuyd (METCZ20190006). NL7594 registred at https://www.trialregister.nl/.

**Supplementary Information:**

The online version contains supplementary material available at 10.1186/s40900-021-00287-w.

## Plain English summary

Exploring care-users experiences is important for decisions to improve quality of care. This applies to care-users in the disability care in particular, as these care-users are highly dependent on their care professional. Instruments that facilitate a dialogue between care-users and care professionals about experiences with care are not always used correctly. Furthermore, it is difficult to translate outcomes into decisions about improving quality of care for the individual care-user and the organisation. In our study, care-users, care professionals, management and researchers developed strategies together to improve the use of care-user experience measures.

This study aims to show the impact of all participants, including care-users, professionals, management and researchers, on developing implementation strategies. Additionally, the study aims to show how care-users can participate in developing strategies whilst having problems with communication due to intellectual, developmental and acquired disabilities.

We found that care-users gave crucial input to the look and feel, and understandability and relevance of the strategies. The contribution of the professionals had impact on how to use strategies and terminology used in instructions and visuals. Management shared lessons learned and represented the needs on the policy level. Researchers used their analytical skills and facilitated the group process. Care-users were able to collaborate by taking into account their needs and because information was presented to them clearly and attractively.

**Supplementary Information:**

The online version contains supplementary material available at 10.1186/s40900-021-00287-w.

## Background

In the Netherlands, around 10,000 people live with disabilities in supported living arrangements as a result of acquired brain injuries (ABI) [1]. They are highly dependent on their professionals. The majority of people with ABI’s are communication-vulnerable. This means that they experience functional communication difficulties, difficulties in expressing themselves and/or in understanding professionals [2]. These difficulties complicate self-advocacy and communication about experiences with received healthcare services while the exploration and measurement of quality of care from the care-user’s viewpoints are fundamental in the context of supported living arrangements [3, 4].

Patient-reported experience measures (PREMs) uncover care-users’ experiences. PREMs are defined as “a measure of patients perceptions of their personal experiences of the healthcare they have received” [5]. PREM insights are essential to guide strategies for care safety improvements and clinical effectiveness, involvement in decision making and effective professional and care-user relationships [3, 6, 7]. The Dutch disability sector encourages the integrated uptake of PREMs, which means that information retrieved in the PREM is used to enhance deliverance of individual care (micro), to improve care on the organisational level (meso) and to facilitate external reporting (macro) [8]. The process of PREM implementation needs to be suitable and tailored to all end-users, from care-users and professionals to organisations board members [9]. Foster et al. (2018) showed that time and resources to design the process (i.e., planning how data will be managed and used) and preparation of the organisations for the implementation (i.e., training professionals) are essential [10].

Implementation strategies that have been co-created with all relevant stakeholders have been found to increase the success of intervention use, because stakeholders add knowledge regarding clinical practice, care-user and professional behaviours, and mechanisms within the organisation [11–14]. However, information about how stakeholders were engaged and what their impact was on strategies is rarely reported [15–18] . Only 56% of the articles from Concannon’s (2014) systematic review reported how stakeholders’ views were synthesised, and only 20% of the articles reported about the impact of engagement. Research using co-creation and reporting about impact only report about professionals and management impact (e.g., improved relevance of the research and research adoption), however, this is often very limited, and the impact of both care-users and researchers is often neglected [17, 18]. Currently, knowledge is lacking about the impact of co-creating implementation strategies with all relevant stakeholders, especially with regard to care-users who are communication vulnerable [15, 19–21].

Involving care-users who are communication vulnerable as stakeholders in developing implementation strategies can be challenging. Information is scarce on activities and tools to engage care-users in a valuable and continuous matter [4, 22]. A structured literature review on the engagement of people with intellectual disabilities in intervention development showed various barriers being experienced [13]. These barriers referred to the preparation phase (recruitment and informed consent), implementation phase (communication, overburdening and challenges in dividing the decision making power) and concluding phase (where the quality of the research outcomes was more important than the process of involving care-users) [13]. These experienced barriers call for supportive conditions to meaningfully engage care-users with ABI’s. People with ABI’s experience problems like aphasia and memory loss, psychological and behavioural problems and physical problems such as epilepsy and fatigue [4, 23, 24]. We define supportive conditions as activities or tools used before, during or after the co-creating meetings that aim to facilitate care-user involvement.

This study presents the third phase of a four-year research project with the overall aim to improve the implementation of a narrative PREM and the integrated use of the outcomes. Strategies are developed with the help of care-users, professionals, and management in the disability sector. This paper aims to provide more insights into the impact of all these stakeholders in developing the implementation strategies, and how the engagement of care-users who are communication vulnerable can be supported. The research questions were:

- What is the impact of the different stakeholders on the developed implementation strategies?
- What supportive conditions facilitate care-users who are communication vulnerable in the development of tailored implementation strategies?

## Methods

### Design

**Fig. 1:**
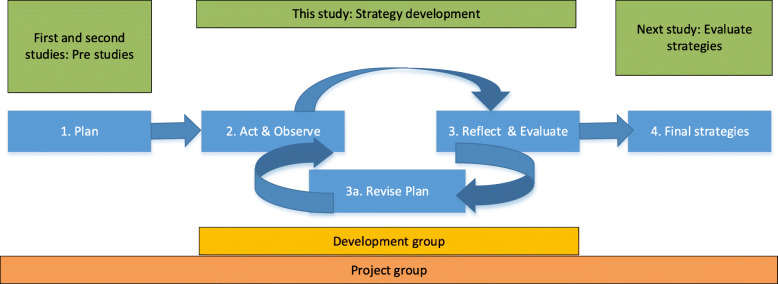
Study design based on participatory action research visualising the project group and development group engagement

We performed a qualitative descriptive design using participatory action research (PAR) to develop implementation strategies in co-creation with relevant stakeholders across organisational levels. PAR involves four iterative self-reflective steps: 1) plan, 2) act and observe, 3) reflect and evaluate, 3a) revise the plan, and 4) implement the revised plan [25]. Our focus is on the role and impact of stakeholders during phase 2 and 3 (see Fig. 1), rather than on the final strategies.

### Setting

We conducted the study at the Stichting Gehandicaptenzorg Limburg (SGL) (Disability Care Foundation Limburg, in English), a care organisation in the south of the Netherlands providing supported living and living arrangements to people with severe acquired, intellectual and developmental disabilities, mostly people with acquired brain injuries (ABI). SGL has worked with the PREM “Dit vind ik ervan!” (DVIE) (“This is how I feel about it!” in English) since 2016. DVIE measures experiences with quality of care by means of a dialogue between the care-user and a professional, and has shown the ability to discover the care-user’s story and experiences [26, 27]. Table 1 provides additional information about the PREM. SGL encountered challenges with the PREM implementation, i.e., embedding the PREM in daily practice and to enable integrated use of the outcomes on the organisational level. Therefore, in 2017, a Project Group was composed to co-create strategies to improve the uptake of the PREM. For more information about the Project Group, see section 2.3.1. The Project Group first identified challenges using a problem analysis, e.g., care-users and professionals did not understand the PREM’s goal, the PREM was not embedded into routine care, and working with PREM outcomes was limited as described in “Implementation of a Patient Reported Experience Measure in a Dutch disability care organisation: a qualitative study “ [29].In a second study, “Stakeholder engagement from problem analysis to implementation strategies for a patient-reported experience measure: A qualitative study on the process and experiences”, the Project Group prioritised these challenges, and designed draft strategies [30]. The two pre-studies formed the plan-phase of the PAR cycle. After this study, the strategy development, a next study evaluating strategies will follow, as shown in Fig. 1.

**Table 1:** Additional information “Dit vind ik ervan!”

“Dit vind ik ervan!” (DVIE) *(This is how I feel about it! In English)* is a measurement that uses an investigating dialogue between the care-user and professional to explore experiences with received care [27]. DVIE is developed bottom-up as a practice based approach by disability care organizations in the Netherlands. During the dialogue ten themes derived from the domains of Schalock can be discussed; feelings, body, family, friends, participation, help, house, doing, decisions and feeling safe [28]. DVIE starts with the dialogue which enables to reach the full scope of the experiences of quality of life. During the dialogue, a set of visual cards can be used to explore experiences with the ten themes. Then care-users rank their experience on a five-point-scale from bad to perfect and indicate if they like a change. In a two-day (10 hours) training professionals are instructed to use DVIE.	

### Participants

Four different types of stakeholders participated: care-users (*n* = 8), professionals (n = 8), management (*n* = 4) and researchers (*n* = 5). The inclusion criteria for participation were:

- Care-users: having an acquired brain injury, being a care-user receiving care from SGL and having interest in the study subject.
- Professionals: having 3 years working experience, experienced in using DVIE (at least once) and able to reflect on their experiences concerning the implementation strategies.
- Management: having 1 year of working experience as a manager, being involved in the application of DVIE at care locations and able to reflect on their experiences concerning the implementation strategies.
- Researchers: having expertise in the field of acquired brain injuries and goal-oriented measuring, and able to reflect on their experiences concerning the implementation strategies.

The exclusion criteria for all stakeholder groups were being unable to communicate verbally or unable to attend meetings physically. We recruited participants by means of purposive sampling to capture a variety of perspectives. A contact person within SGL approached and provided team leaders and professionals with verbal information and a visual summary of the study. Team leaders and professionals invited the care-users.

Participants were already involved in the “Project Group” in the plan phase. Both groups had a different focus. They alternated each other.

#### Project group

The Project Group is engaged from the beginning of the project in 2017 and represents all stakeholders throughout the organisation. The Project Group has been engaged in the problem analysis [29] and in drafting the implementation plan according to the identified barriers [30]. In this study the focus of the Project Group was on step 2 (act and observe), step 3 (reflect and evaluate), and step 3a (revising the draft strategies). Additional file 1 shows details about stakeholder type, relevance for engagement, sex and educational level of the participants of the Project Group.

#### Development groups

Two Development Groups (group A and B) were composed in order to test and evaluate draft implementation strategies, in step 2 (act and observe) and step 3 (reflect and evaluate), using co-creation methods. Additional file 2 shows the role, relevance for engagement, sex and educational level of the participants of the Development Groups.

Figure 1 shows the study design based on participatory action research.

A junior researcher (MvR) led all meetings of the Project Group and Development Groups. Her role was to facilitate engagement of all stakeholders during the meetings, and to send a short notes to report all agreements, decisive moments and necessary actions after the meetings. Besides these reports, the junior researcher took pre- and post-development meeting field notes to reflect on interactions, behaviour and non-verbal communication during the co-creative meetings. In addition, she had regular informal conversations with individual participants in between meetings to reflect upon or prepare for the next meeting. The junior researcher contacted participants who could not attend a meeting in order to ask for their feedback on the strategies discussed.

### Ethics

Before inclusion, participants received written and verbal information about the study. After inclusion, one of the researchers visited all care-users individually to explain the goal of the meetings. All participants could ask questions prior to giving informed consent and during the meetings. They were given enough time to ask questions and were free to indicate if they wanted to stop or needed a break. We guaranteed confidential and anonymous handling of data by using codes. The study was reviewed by the Ethics Committee of Zuyderland-Zuyd (METCZ20190006).

### Data collection and analysis

The study took place in nine months from May 2019 to January 2020. The Project Group and Development Groups met on a regular basis, 5 and 13 times, respectively. Data collection was ongoing and consisted of 18 summaries of protocols preparing group meetings, 18 reports and 19 audiotapes of group meetings (60–90 min), and 33 notes of verbal and written informal conversations with the junior researcher and stakeholders of the Project and Development Groups.

Data were analysed using conventional content analysis by two researchers [31]. The analysis resulted in two categories and 10 subcategories for the research question regarding the impact of stakeholders, and 4 categories and 12 subcategories for the research question regarding the communication-supportive strategies.

### Trustworthiness

We used several strategies to enhance trustworthiness. Credibility was enhanced by prolonged engagement, since time was invested in becoming familiar with the setting and the junior researcher stayed in touch with all stakeholders, using email and individual visits to care-users throughout the process, to test for misinformation and to build trust with the stakeholders. Investigator triangulation was secured using extensive iterative peer debriefing of the research process and analysis, by means of discussions with the research team and the Development Groups. Source triangulation was secured by involving care-users, professionals, management and researchers. Method triangulation was secured by using protocols, reports, audiotapes and researchers’ notes. All reports were member-checked by starting each meeting with a quick summery of topics discussed in the report. Transferability was secured by thick description of the setting, research process and methods used [32].

## Results

The results first present the impact of the different stakeholders on the developed implementation strategies. Next, findings are shown regarding supportive conditions that facilitate care-users who are communication-vulnerable in the development of tailored implementation strategies. In order to provide context to the results, we show the final strategies to improve the integrated PREM uptake in supplementary file 1. These strategies aim to diagnose the state of PREM use (QuickScans and Learning goal meeting), inform participants about the PREMs goal, added value, execution and working with outcomes (Kick-off, Film, Infographic, Pocket booklet, Process description and Team reflection), and teach care professionals skills about PREM use (Refresher training and Coaching on the job). The set of strategies therewith tries to first change peoples’ actions and as a result change practice. A reporting checklist following the Guidance for Reporting Involvement of Patients and Public (GRIPP2) Short Form can be found in Supplementary Materials 2 [33].

### Impact of stakeholders on implementation strategies

We present our analysis of the stakeholders’ impact on the implementation strategies per stakeholder group. Themes are shown in Table 2.

**Table 2:** The impact of engaged stakeholders

Stakeholder	Theme
Care-users	Look and feel
	Understandability and relevance
Professionals	How to use the strategies?
	Terminology use
Management	Gap between policy and practice
	Learning from previous improvement failures
Researchers	Analysis and direction of strategy changes
	Translating academic and development experience into practice

#### Care-users

In this section, we describe the results of care-users’ impact on the final strategies, categorised as look and feel, and understandability and relevance.

##### Look and feel

**Fig. 2:**
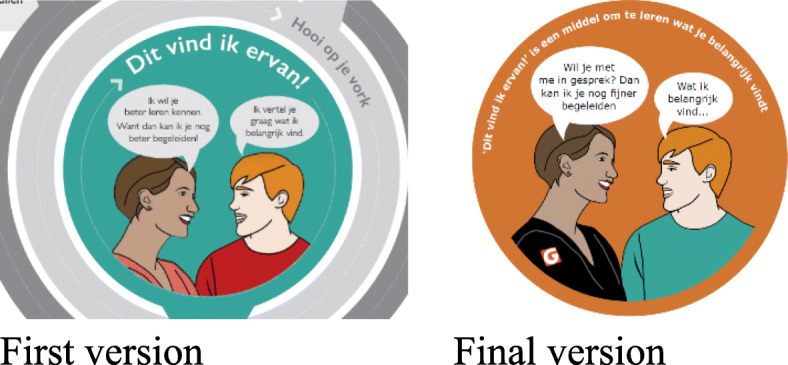
The infographic

Care-users had an impact on the look and feel of strategies to improve strategies’ visibility and use. Some care-users pointed out that they could not see grey or taupe colours, potentially due to their ABI. Therefore, we changed colours into contrasting and brighter colours like black, orange or blue. Moreover, care-users helped clarify the visuals or figures, for example in the infographic (see Fig. 2). In the *infographic*, the two figures represent a care-user and professional. Care-users did not understand who was who, and suggested to use the ‘G’ out of the organisation’s logo ‘SGL’ on the shirt of the professional.

##### Understandability and relevance

Testing strategies provided insight into the potential use of the strategies. For example with testing the *QuickScan,* a questionnaire exploring the implementation status at a facility. Care-users in the Development Group experienced the *QuickScan* as too complex. Some care-users’ needed a reading aid because of ABI related memory problems. They wanted a leaflet as an introduction to the *QuickScan*. Others asked their case-manager for help, which influenced the answers’ objectivity. In the leaflet, we added the advice to ask an independent professional for help to fill out the *QuickScan.*

In addition, care-users improved the relevance of the implementation strategies. For example, in the draft-*QuickScan,* all stakeholders were asked if they understood the goal of all types of measurements used in the organisation. Care-users felt they did not have to know the goal of all measurements, making this question irrelevant for them to answer. Moreover, care-users did not want many pages (max. 10) in the *Pocket booklet,* developed to enable care-users to prepare themselves for the PREM. Care-users liked having limited decision options in the *QuickScan* to prevent confusion; yes or no instead of good, moderate, poor.

*Care-user3: It would have been much clearer if I could answer with “Yes” or “No”. Then you’d directly answer the question. Because what is ‘moderate’. I think ‘moderate’ is a rather elastic concept.*

Moreover, care-users preferred Dutch names instead of the draft-strategies’ English names, such as *Infographic*. Therefore, we changed *infographic* into *information poster* (*informatie poster).* Care-users wanted abbreviations to be spelled out and preferred words they would recognise and regularly use in their care organisation. For example, in the *QuickScan*, instead of talking about *“your location” (jouw locatie)*, they preferred the word *“your community” (jouw woongroep)*.

#### Professionals

The impact of professionals was on clarifying guidance in the strategies’ use and PREM specific terminology.

##### How to use the strategies?

Professionals experienced problems to follow instructions in both the *Pocket booklet* and *QuickScan,* and asked the researcher for clarification. They wanted a step-by-step guidance, first describing the goal, then how the strategy should be used and what should be done to complete the strategy. Preferably described in short sentences and presented in bullet points.

Testing strategies showed the strategies’ potential shortcomings in practice for professionals. For example, in the *QuickScan,* a statement focussed on if professionals knew the difference between measurements used in the organisation. While filling out the question, a professional asked for clarification from a colleague because she did not know the difference between the PREM and other measurements used. This identified the need for clear instructions and that professionals should fill out the *QuickScan* solo.

##### Terminology use

Professionals, who were also working as PREM trainers, helped to align phrases when talking about the PREM. For example, in the *Pocket booklet,* the draft version used “conversation” to represent the PREM execution, whereas trainers felt it should be “dialogue”, since that word is most often used within the PREM ideology. Moreover, researchers sometimes interpreted words differently compared to professionals. For example, professionals experienced the word *“better”* in the *QuickScan* as if things were not good at all. They wanted constructive words like “*improve”*.

#### Management

The impact of management was on showing the gap between management policy and practice, and learning from previous improvement failures.

##### The gap between policy and practice

Developing the strategies sometimes resulted in discussions about the structure and vision of the organisation. These discussions demonstrated challenges between knowledge about policy made by management, and professionals working according to the policy. There were differences in both the goal and connotations regarding the PREM and other measurements used within the organisation. To overcome this gap, the *Process description* was co-created, visualising when the different measurements had to be used within the yearly care cycle.

##### Learning from previous improvement failures

Management shared experiences with previous unsuccessful initiatives to improve the PREM implementation. For example, in the past, they learned that no participants signed up for a non-obligatory *coaching on the job*. Based on this experience, the group decided to make the *coaching on the job* for both professionals and team leaders mandatory.

#### Researchers

The impact of the researchers was on the analysis and direction of strategy changes, and translating academic and development experience into practice.

##### Analysis and direction of strategy changes

Researchers had an impact on the analysis of feedback and deciding what changes were made in the strategies. After each meeting, outcomes of the tested strategies and evaluative points were reported in a way that all stakeholders were able to follow the process without being overloaded with information. Researchers were in the lead of analysing outcomes of meetings and responsible for communication about outcomes with all stakeholders. The researchers decided what would be the next steps within the process together with the Project Group.

##### Translating academic and development experience into practice

Researchers used their experiences of previous intervention development and translated research knowledge into practice. For example, a researcher with expertise in communication-vulnerability helped to fine-tune a spacious lay-out and making text easy to understand for *the Pocket booklet*. Moreover, a researcher that was expert in co-creation and a Professor in effective measuring emphasised the importance of *key players* in participating locations to spread the responsibility of the PREM implementation process over the different stakeholders.

### Supportive conditions

We present our analysis of supportive conditions for care-users who are communication vulnerable using two themes: organisational issues of meetings and presentation of information. Themes and subthemes are shown in Table 3.

**Table 3:** Themes and subthemes of supportive conditions

Supportive conditions	
Organisational issues of the meetings	Timing of meetings
	Repetitively explaining
	Sharing ownership
	Cherishing relationships and a safe environment
Presentation of information	Form
	Language
	Content
	Meeting report

#### Organizational issues of the meetings

##### Timing of meetings

Before the start of the project, the researcher inquired with care-users when their energy levels were highest during the day and organised meetings at times most convenient for them. Several care-users suffered from loss of energy during the day, therefore, one group preferred to have a meeting at 10.30 a.m., before lunch and not too early, and the other group preferred 1.00 p.m. because they felt energised right after lunch.

##### Repetitively explaining

For care-users, it was challenging to grasp the project’s goal. In the first meeting, some care-users thought they had to develop a new PREM, or they started talking about their personal experiences with quality of care, instead of testing and discussing the draft-strategies. For this reason, at the beginning of every meeting, we explained the meeting’s goal and repetitively explained the goal of the project to ensure we started with a common understanding. However, although care-users at times did not understand the project or meeting goal, they still had valuable input in discussing concrete aspects of strategies such as colour and word choices.

##### Sharing ownership

At the start and end of each meeting, the researcher explicitly gave care-users the opportunity to come forth with their ideas or wishes for changes in how meetings were done. For example, care-users wanted a quicker follow up on strategy feedback and preferred to discuss just one draft-strategy per meeting.

##### Cherishing relationships and a safe environment

Researchers put effort into building a cherishing relationship and a safe environment with care-users. The participation of a care-user in both the Project Group and the Development Groups was valuable. A team leader stated:

*Team leader: “I like that she (care-user participating in both Project and Development Group) is part of the group, as she has two different roles. I think this helps care-users in feeling safe to share their experiences and thoughts, which then provides us with a view into what’s important for them.”*

Care-users wanted individual meetings with the researcher to get to know each other. This helped to build a relationship and offered opportunities to clarify information, ask questions and signing the informed consent. In addition, the researcher used these meetings to have a more detailed view of the care-users on strategies, e.g., formulating specific statements in the *QuickScan*. Moreover, during the meetings of the Project and Development Groups, the researcher created a safe environment by providing space for all stakeholders to share their opinions, and letting care-users complete their sentences without interruption. Additionally, Development Groups had equal numbers of care-users and professionals to improve a safer environment. Meetings had a non-obligatory character and care-users were given the feeling that their handicap did not make a difference.

*Care-user: “Because of these groups, I dare to speak up more, I dare to go outside and talk about how I feel about things… In other situations, people do not let me finish, or I feel they do not listen to what I say because they are not interested… People think oooh she’s disabled, she just needs help and talks nonsense. But here I feel that you are really listening.”*

#### Presentation of information

##### Form

The researcher experimented with different types of presentations: paper handouts, using PowerPoint presentations and sharing verbal information only. Some care-users had difficulties holding a handout because of stiff joints that complicated turning over pages. Care-users preferred verbal presentations supported by a PowerPoint to help visualise what was being talked about. Physical examples or simple pilots enabled care-users to provide feedback on strategy ideas. An example of a PowerPoint presentation is found in Supplementary Materials 3.

##### Language

Care-users requested the researcher to use easy language, no long sentences or distinguished wording, and to keep it simple by using high frequency and high imaginable words. One care-user explained that people using difficult words made her feel insecure to express her thoughts.

*Care-user: ‘During these meetings, it is simple, but outside on the streets, people do use difficult words which makes me feel insecure. I do not dare to speak, because I feel judged. They might think I am crazy for not understanding them….”*

##### Content

At the beginning of the project, care-users experienced information overload. As a result, they lost focus or interchanged strategies. Because care-users only wanted to be involved in designing strategies that were directly related to them, we limited the number of topics per meeting. Once we split the Development Group into a care-users group and a professionals group In this meeting, professionals focused on the *process description*. With the care-users, we discussed what supported them during the meetings.

##### Meeting report

After each meeting, meeting reports were made with communication-supportive techniques, such as a structured lay-out, using 1.5 line spacing, a lot of white space, short sentences (max. 10 words per sentence), high frequency words, bolding key concepts and one message per sentence. We also used visual strategies, such as bright colours, drawings, photos, picto’s and smileys. Care-users experienced these meeting reports as helpful to remember the previous meetings.

## Discussion

This study aimed to provide more insights into the impact of all relevant stakeholders, including communication-vulnerable care-users, in co-creating implementation strategies, and how these communication-vulnerable care-users can be supported to be engaged. Our analysis illustrated the impact of care-users on the strategy’s look and feel, and understandability and relevance. Professionals created an impact on how to use the strategies and on the terminology used. Management showed the gap between policy and practice, and shared experiences with previous improvement failures. The researchers had an impact on analysis, the direction of strategy changes and translating academic and development experience into practice. The engagement of care-users was supported by taking into account organisational issues such as timing and cherishing relationships, and presentation of information using easy language and visual reports.

The study showed a complementary contribution of all the different and relevant stakeholders. Engaging all stakeholders resulted in more clarity regarding the policy behind the PREM and the policy–practice gap that exists. Care-users and professionals had an impact on a practical level by testing strategies and providing feedback for strategies’ improvements. Without their input, colours would not have been visible, and strategies were at risk of “information overload” for the care-users, e.g., in the *pocket booklet* and *infographic*. The care-users who participated in our Development Groups were mainly from a low educational background. Therefore, their specific needs to be actively involved in the Project Group, and their impact on the strategies may not be only a result of their ABIs but also from their intellectual skills. However, it was not the scope of our study to provide data saturation about the needs of people with ABI to be engaged in stakeholder meetings. In our groups, all the participants were able to express adjustments they wanted to see in the strategies. Management provided a complimentary “helicopter view”, showing what policy ought to be, but also provided insight into earlier failed improvement initiatives that prevented the strategies from making similar mistakes. Michaelson (2016) showed similar findings in her study adapting an intervention for a paediatric intensive care unit. The impact in her study was identified mainly through piloting, which resulted in a navigator-guide co-created with the end-users [18]. Our study confirms this challenge of translating policy into practical guidelines and underlines the importance of stakeholder engagement to impact the guidance needed for care-users and professionals using the strategies.

However, not many studies have yet described the impact of researchers in the process of stakeholder engagement (Majid et al. 2018; Michelson et al. 2016), even though their role cannot be neglected. While representing a broad variety of expertise (supporting people who are communication-vulnerable, designing and implementing complex interventions, and behaviour change) they were responsible for design and content decisions based on the care-users, professionals and management input. Lofman (2004) and Scheffelaar (2020) emphasised the possible unequal relationships between the researchers and care-users and the importance of relationship-building to overcome this issue [34, 35]. Our study showed that, especially for the care-users, cherishing relationships and creating a safe environment enabled this group to feel supported to participate. We organized researcher care-user meetings to introduce care-users to the project, stayed in touch in between meetings and adapted meetings according to care-users wishes which made care-users feel listened to. Nevertheless, the engagement of care-users demanded flexibility and creativity in organising meetings. Sometimes we needed to change the plan during meetings. For example, when we discussed the process description with Development Group, care-users did not see the relevance for them, whereas management and professionals did feel the importance of clarifying the PREM process. Therefore, the researchers decided to split the Project Group. Both the care-users and the other stakeholders positively experienced splitting the group. Frankena (2015) and Shippee (2015) underwrite these findings. They showed that being flexible and changing the form, time or accommodation does sometimes cost extra time and energy but is an unavoidable issue researchers should be prepared to take [13, 14]. However, in the evaluation phase of a participatory action research, it is important that researchers do no longer have influence on the implementation process. Then, they solely take on an observing role to provide a more objective view upon how strategies are used in practice.

In our study, we have shown that care-user engagement can be more than tokenism. The care-user engagement in both the Project and Development Groups created significant changes because care-users continuously participated and it even resulted in personal growth for the care-user. During the meetings, care-users’ ownership of the strategies increased and they enjoyed being engaged. Care-users experienced that their skills in speaking up and discussing topics improved, which are aspects confirmed in earlier research [14, 36]. Moreover, the engagement of all stakeholders increased awareness and enthusiasm to improve implementation of the PREM. McDonald’s (2016) empirical study on co-creation with people with disabilities, as well as the systematic review of Shippee (2015) on care-user engaged research, showed similar outcomes. They underlined the importance of sharing leadership, by showing what is done with care-users’ input and amplifying the shared decisive role for the care-users [14, 36]. The feeling of sharing leadership was increased by having an experienced care-user who participated in both a Project and Development group. Because of the care-users, they had a voice in the agenda-setting from the start of the project. Seeing peers engage has been proven to benefit the strategy uptake and integration [36, 37].

The early engagement of the Project group, in all phases of the PAR, provided in-depth information on the engaged disability care organisation but also enabled the Project Groups’ impact on decision making from the start. This close collaboration with all stakeholders has potential to improve later collaboration between researchers and organisations’ stakeholders. The collaboration helps to steer the agenda and provides context. Thereby, a relevant perspective is ensured and care-users are prevented from being relegated to a disempowered subject with no impact [14].

However, to support this collaboration, we invested a lot of time in designing strategies that fit with all stakeholder types to deliver a strategy plan enabling integrated PREM use. The intense and constructive collaboration with the care-users enabled the design of a tailored PREM strategy process. We cannot claim that this intense collaboration process results in an effective implementation strategy. Therefore, a process evaluation is planned to get insight into experiences and use of the strategies and the success of the implementation in terms of integrated PREM use.

### Methodological reflection

A methodological strength was the use of PAR, a frequently used approach within intervention development engaging stakeholder, which provided a systematic approach in co-creating the strategies [25].

This study was also subject to some limitations. The directive role of researchers potentially resulted in researcher bias, because the analysis was done with the same research team that was engaged during the study. It benefitted the understanding of the process because researchers had been engaged in every step. However, the researchers’ objective views on the process were potentially biased due to their prolonged engagement in the process, resulting in a culture bias [38]. Moreover, because care-users were selected by a professional, selection bias was possible. Research shows that professionals tend to select the most accessible care-users, a limitation not uncommon but difficult to overcome in stakeholder engagement in this sector [11, 13, 32]. Even though we strived for an inclusive representation of all relevant stakeholders, the Project Group care-user had to be able to communicate verbally to participate in discussions on a more abstract level for micro, meso and macro-level strategies. In the Development Group, the focus was only on micro-level strategies. This enabled care-users to participate that could only communicate verbally, e.g., when deciding about colours or visuals.

## Conclusion

Extra efforts to enable the engagement of all relevant stakeholders are crucial in the process of developing implementation strategies for a PREM. The impact of care-users who are communication vulnerable concerned the strategies’ look and feel, and understandability and relevance. Professionals created an impact on how to use the strategies and terminology. The effect of management was on showing the gap between policy and practice, and learning from previous improvement failures. Researchers showed an impact on analysis, direction of strategy changes and translating academic and development experience into practice. Second, this study showed conditions that supported the engagement of care-users who are communication vulnerable. Taking into account organisational issues and presentation of information, facilitated their impact on the implementation strategies.

## Supplementary Information

**Additional file 1: Appendix 1.** Participants of the Project Group

**Additional file 2: Appendix 2.** Participants of the Development Groups

**Additional file 3: Supplementary Materials 1**. Implementation Strategies

**Additional file 4: Supplementary Materials 2**. A reporting checklist following the Guidance for Reporting Involvement of Patients and Public (GRIPP2) Short Form

**Additional file 5: Supplementary Materials 3.** An example of a PowerPoint presentation

## Data Availability

The authors confirm that the data supporting the findings of this study are available within the article [and/or] its supplementary materials.

## Abbreviations

ABI: Acquired brain injuries
DVIE: Dit vind ik ervan!
PAR: Participatory action research
PREM: Patient-reported experience measure
