# Supplementary material for: How to foster successful implementation of a patient reported experience measurement in the disability sector: an example of developing strategies in co-creation
Source: Res Involv Engagem. 2021 Jun 24;7:45. doi: 10.1186/s40900-021-00287-w (PMC8229276; doi:10.1186/s40900-021-00287-w)
Supplement: Supplementary file 1 — Additional file 1: Appendix 1. Participants of the Project Group [file 40900_2021_287_MOESM1_ESM.docx]

**Appendix 1** Participants of the Project Group

| **Stakeholder type** | **Relevance for engagement** | **Sex** | **Educational level** |
| --- | --- | --- | --- |
| Care-user, experienced as care-user representative | Care-user experienced with low energy levels and stuttering | F | Primary education |
| Professional | PREM trainer | F | Tertiary education |
| Professional | PREM trainer | F | Tertiary education |
| Management employee | Team leader | M | Higher tertiary education |
| Management employee | General quality manager | M | Master |
| Management employee | Quality of care advisor | F | Master |
| Management employee | Psychologist and education advisor | F | Master |
| Researcher | Professor in goal-oriented measurement | F | Doctoral |
| Researcher | Senior researcher expert in co-creation and qualitative research | F | Doctoral |
| Researcher | Senior researcher expert in communication vulnerability | F | Doctoral |
| Researcher | Senior researcher expert in co-creation and goal-oriented measurement | F | Doctoral |
| Researcher | Junior researcher in health sciences and project leader | F | Master |
