# Supplementary material for: How to foster successful implementation of a patient reported experience measurement in the disability sector: an example of developing strategies in co-creation
Source: Res Involv Engagem. 2021 Jun 24;7:45. doi: 10.1186/s40900-021-00287-w (PMC8229276; doi:10.1186/s40900-021-00287-w)
Supplement: Supplementary file 2 — Additional file 2: Appendix 2. Participants of the Development Groups [file 40900_2021_287_MOESM2_ESM.docx]

**Appendix 2.** Participants of the Development Groups

| **Role** | **Relevance for engagement** | **Sex** | **Educational level** |
| --- | --- | --- | --- |
| Group A | | |  |
| Care-user | Care-user experienced with autism and low energy levels | M | Primary education |
| Care-user | Care-user experienced with quick attention loss, social-emotional vulnerability and low energy levels | F | Secondary education |
| Care-user and member of the Project Group | Care-user experienced with low energy levels and stuttering | F | Primary education |
| Professional | Care provider and PREM trainer | F | Tertiary education |
| Professional | Care provider and locations communications advisor | F | Secondary education |
| Professional | Managing location | M | Tertiary education |
| Group B | | |  |
| Care-user | Care-user experienced with deafness and difficulties following conversations | F | Secondary education |
| Care-user | Care-user experienced with quick attention loss and difficulties following conversations | M | Primary education |
| Care-user | Care-user experienced with quick attention loss and low energy levels | M | Secondary education |
| Care-user | Care-user experience with difficulties following conversations and stuttering | F | Primary education |
| Professional | Care provider | F | Secondary education |
| Team leader | Managing location | F | Tertiary education |
