# Supplementary material for: How to foster successful implementation of a patient reported experience measurement in the disability sector: an example of developing strategies in co-creation
Source: Res Involv Engagem. 2021 Jun 24;7:45. doi: 10.1186/s40900-021-00287-w (PMC8229276; doi:10.1186/s40900-021-00287-w)
Supplement: Supplementary file 3 — Additional file 3: Supplementary Materials 1. Implementation Strategies [file 40900_2021_287_MOESM3_ESM.docx]

| **Implementation Strategies** | |
| --- | --- |
| QuickScan | Questionnaire to explore the state of working with the PREM at the start (T0) and the end (T1) of the implementation process. QuickScans are tailored to stakeholders’ scope of interest. Questions for care-users differ from questions for professionals and managers. |
| Learning goal meeting | Meetings to formulate facility-specific learning goals according to QuickScan results and a learning goals guide. Participants are a care-user, professionals and a manager. |
| Kick-off | Session to introduce the PREM (by a film and infographic) and to share facilities learning goals. Participants are all care-users, professionals, facility manager and the care-user representative. |
| Film | Short figurative story to explain the added value of the PREM, <https://www.youtube.com/watch?v=hCsRuv3Bz1g&t=8s>. It is presented to the participants of the kick-off session. |
| Infographics | Illustration to explain the goal of the PREM goal (for care-users) or to explain both the PREM goal and its relation to other measurements (for professionals and facility managers). |
| Pocket booklet | A6 booklet for care-users to help prepare, execute and reflect on PREM dialogue. Provided by professionals to all care-users. |
| Process description | Illustration that explains PREM integration in the annual cycle of care. It is provided to all professionals and facility managers. |
| Refresher training | Two hour training session addressing location specific learning goals and discussing PREMs added value, the process (using process description) and how it works in practice (using pocket booklet and addressing communication supportive tools such as talking mats, picto’s and pen and paper). Provided to all professionals by a colleague who is certified PREM trainer. |
| Coaching on the job | Observation of a dialogue between a trained professional and a care user to provide feedback. The certified PREM trainer provides this coaching on the job. |
| Team reflection | Team reflection on PREM execution and/or outcomes and formulation of potential actions for facilities. Participants of these sessions are the professionals and facility manager (sessions per facility). |
| **Process of using the implementation strategies** | |
|  | |

*Supplementary materials 1: Overview of the implementation strategies.*
