# Supplementary material for: How to foster successful implementation of a patient reported experience measurement in the disability sector: an example of developing strategies in co-creation
Source: Res Involv Engagem. 2021 Jun 24;7:45. doi: 10.1186/s40900-021-00287-w (PMC8229276; doi:10.1186/s40900-021-00287-w)
Supplement: Supplementary file 4 — Additional file 4: Supplementary Materials 2. A reporting checklist following the Guidance for Reporting Involvement of Patients and Public (GRIPP2) Short Form [file 40900_2021_287_MOESM4_ESM.docx]

| **Section and topic** | **Item** | **Reported on page No** |
| --- | --- | --- |
| 1: Aim | Report the aim of PPI in the study  *Using the experience and knowledge from care-users, professionals and management employees to develop implementation strategies for a Patient-Reported Experience Measure in the disability sector.* | P. 8-9 |
| 2: Methods | Provide a clear description of the methods used for PPI in the study  *PPI was facilitated by means of co-creation sessions in development group meetings and in the project group. In development group meetings, care-users, professionals and facility managers tested and evaluated draft implementation strategies,*  *The project group aimed to improve the integrated use of the Patient-Reported Experience Measure and included researchers, a care user representative, professionals, and management employees from the care organization for which the implementation strategies were developed. Based on the input from the co-creative sessions within the development groups, they reflected on the implementation strategies, decided which revisions took place for the final implementation plan. The project group members met each other during group sessions, but also contacted each other by mail or phone about minor details of the implementation strategies.* | P. 9 |
| 3: Study results | Outcomes—Report the results of PPI in the study, including both positive and negative outcomes  *Care-users were able to provide valuable and crucial input on lay-out and understandability of implementation strategies, for example font style and size in infographics.*  *We found some organization conditions were important to make sure that the (communication vulnerable) care users were able to participate. The sessions had to be flexible with regard to the specific time during a day, and the duration of the meeting (<1 hour), as they become tired soon. Information needs to be presented in a simple and attractive way, for example reports with visuals and short sentences (10 words max) with one message.*  *The contribution of the care professionals had impact on how to use strategies, and on terminology used in strategy instructions and visuals. Management employees shared lessons learned from previous improvement failures and represented the needs on the policy level. Researchers used their analytical skills and facilitated the group process.* | P.14-17 |
| 4: Discussion and conclusions | Outcomes—Comment on the extent to which PPI influenced the study overall. Describe positive and negative effects  *Positive: PPI highly influenced the study outcomes, which was according to the aim of the study. The early engagement of care users, professionals and management employees in the project group provided in-depth information on the engaged disability care organisation. It also enabled the project groups’ impact on decision making from the start of the study.*  *Negative; Due to our strong focus on PPI, we invested a lot of time in designing strategies that would fit with all stakeholder types in order to deliver an implementation plan for an integrated PREM use.* | *P. 24* |
| 5: Reflections/critical perspective | Comment critically on the study, reflecting on the things that went well and those that did not, so others can learn from this experience  *Our intense and constructive collaboration with the care users enabled the design of a tailored PREM strategy process. Future research could investigate if less intense contact is possible for reaching the same outcomes in terms of care user engagement.*  *Moreover, researchers had a main role by means of organizing analysis and making all reports. This potentially led to researcher bias. Lastly, it is challenging to guarantee a full representation of all care users, since many people in the disability sector do not have the ability to communicate in a verbal way.*  *However, we would definitely recommend future investigators to engage with care-users, as their contribution is indispensable with regard to the look and feel, understandability and relevance of implementation strategies.* | *P. 24-25* |

Supplementary Materials 2: A reporting checklist following the Guidance for Reporting Involvement of Patients and Public (GRIPP2) Short Form (31)
