# Supplementary figures and images for: How to foster successful implementation of a patient reported experience measurement in the disability sector: an example of developing strategies in co-creation
Source: Res Involv Engagem. 2021 Jun 24;7:45. doi: 10.1186/s40900-021-00287-w (PMC8229276; doi:10.1186/s40900-021-00287-w)

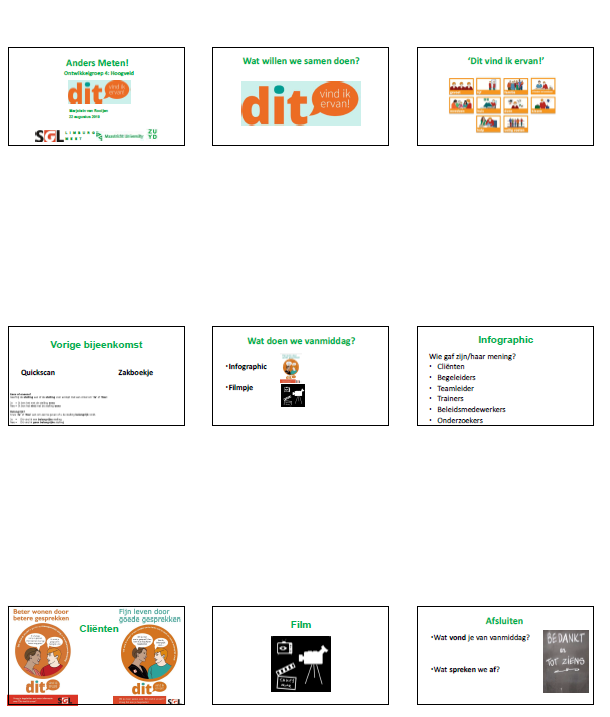


**Supplementary Materials 3:** An example of a PowerPoint presentation

Supplement: Supplementary file 5 — Additional file 5: Supplementary Materials 3. An example of a PowerPoint presentation [file 40900_2021_287_MOESM5_ESM.docx]
